# Composition and risk assessment of perioperative patient safety incidents reported by anesthesiologists from 2009 to 2019: a single‐center retrospective cohort study

**DOI:** 10.1186/s12871-020-01226-0

**Published:** 2021-01-07

**Authors:** Xue Zhang, Shuang Ma, Xueqin Sun, Yuelun Zhang, Weiyun Chen, Qing Chang, Hui Pan, Xiuhua Zhang, Le Shen, Yuguang Huang

**Affiliations:** 1 Department of Anesthesiology, Peking Union Medical College Hospital, Chinese Academy of Medical Sciences and Peking Union Medical College, Shuaifuyuan 1#, Dongcheng District, 100730 Beijing, China; 2 Department of West Campus Medical Affairs, Peking Union Medical College Hospital, Chinese Academy of Medical Sciences and Peking Union Medical College, Damucang Alley 41#, Xicheng District, Beijing, China; 3 Central Research Laboratory, Peking Union Medical College Hospital, Chinese Academy of Medical Sciences and Peking Union Medical College, Shuaifuyuan 1#, Dongcheng District, Beijing, China; 4 Department of Medical Affairs, Peking Union Medical College Hospital, Chinese Academy of Medical Sciences and Peking Union Medical College, Shuaifuyuan 1#, Dongcheng District, Beijing, China

**Keywords:** Anesthesia, Incident reporting system, Patient safety, Risk assessment

## Abstract

**Background:**

Patient safety incident (PSI) reporting has been an important means of improving patient safety and enhancing organizational quality control. Reports of anesthesia-related incidents are of great value for analysis to improve perioperative patient safety. However, the utilization of incident data is far from sufficient, especially in developing countries such as China.

**Methods:**

All PSIs reported by anesthesiologists in a Chinese academic hospital between September 2009 and August 2019 were collected from the incident reporting system. We reviewed the freeform text reports, supplemented with information from the patient medical record system. Composition analysis and risk assessment were performed.

**Results:**

In total, 847 PSIs were voluntarily reported by anesthesiologists during the study period among 452,974 anesthetic procedures, with a reported incidence of 0.17%. Patients with a worse ASA physical status were more likely to be involved in a PSI. The most common type of incident was related to the airway (*N* = 208, 27%), followed by the heart, brain and vascular system (*N* = 99, 13%) and pharmacological incidents (*N* = 79, 10%). Those preventable incidents with extreme or high risk were identified through risk assessment to serve as a reference for the implementation of more standard operating procedures by the department.

**Conclusions:**

This study describes the characteristics of 847 PSIs voluntarily reported by anesthesiologists within eleven years in a Chinese academic hospital. Airway incidents constitute the majority of incidents reported by anesthesiologists. Underreporting is common in China, and the importance of summarizing and utilizing anesthesia incident data should be scrutinized.

## Background

According to the World Health Organization (WHO) international classification for patient safety, a patient safety incident (PSI) is an event or circumstance that could have resulted, or did result, in unnecessary harm to a patient [1]. PSI reporting has been an important means of improving patient safety and enhancing organizational quality control. Many developed countries, such as the United States of America, Australia, the United Kingdom and Germany, have had national PSI reporting systems for prospective collection of PSI data since 1993 [2–6]. For example, hospitals in England and Wales are obligated to report PSIs to the UK National Reporting and Learning Service (NRLS), and data are periodically analyzed at the national level. These systems similarly encourage the blame-free submission of incident reports, with the aim of identifying such defects before causing harm [3].

By understanding the theory more thoroughly and due to the development of new technologies, such as the application of the visual laryngoscope and laryngeal mask, as well as the discovery of new drugs such as sugammadex, anesthesia has become safer in recent decades. However, the quality of anesthesiologists’ work could be challenged by the increasing number of old and sick patients, more complicated surgical procedures, new drugs and new equipment, increasing pressure and professional burnout. The Declaration of Helsinki, as amended in June 2010, emphasized that all institutions providing anesthesia care to patients must contribute to the recognized national or other major audits of safe practice and to critical incident reporting systems [7]. In a 2019 European survey, 78.7% of responders stated that their hospital used a critical incident reporting system [8]. In the United Kingdom, a specialty-specific incident reporting system for anesthesia was introduced in 2010 [9]. However, few studies have characterized incidents from anesthesia practice, and none of these have been from developing countries.

To our knowledge, the nation-wide incident reporting system has not been well managed in China. It has become a significant waste of clinical information because only timely identification of errors makes pre-emptive efforts for clinical change and improvement possible. As the top hospital in China, Peking Union Medical College (PUMC) Hospital established a PSI reporting system in 2009. In the present study, we analyzed all PSIs reported by anesthesiologists in PUMC Hospital in an eleven-year period to share information with other anesthesiologists to better improve patient safety in perioperative care.

## Methods

### Data collection

An incident reporting system was established in 2009 in PUMC Hospital. All healthcare workers are authorized to log into the system and report PSIs either anonymously or not. The incidents were described in freeform text to provide information on patient circumstances, details of the incident, perceived contributing factors, hidden dangers, and suggestions for prevention. The database was examined for all incidents reported by anesthesiologists from September 2009 to August 2019 with the approval of the Peking Union Medical College Hospital Institutional Review Board (S-K1107, 25 March 2020). Data were also collected from the patients’ records, including anesthesia records, to supplement the information in the incident reports.

### Data processing

The incidents were evaluated by reviewers for processing. Eight reviewers from the department of Anesthesiology consolidated the data into an Excel format. All members of the research group signed confidentiality agreements before receiving the data. To ensure validity and reliability throughout the study, all the members received uniform training about the data extraction. Eight reviewers were divided into four groups, with two reviewers in each group. Two reviewers reviewed the same part of the data separately, and they met to discuss discrepancies until they reached an agreement. When discrepancies could not be resolved by discussion between the two reviewers, the problem was discussed at a weekly meeting of the entire research group under the direction of the senior investigators (Professor YH and LS).

**Fig. 1:**
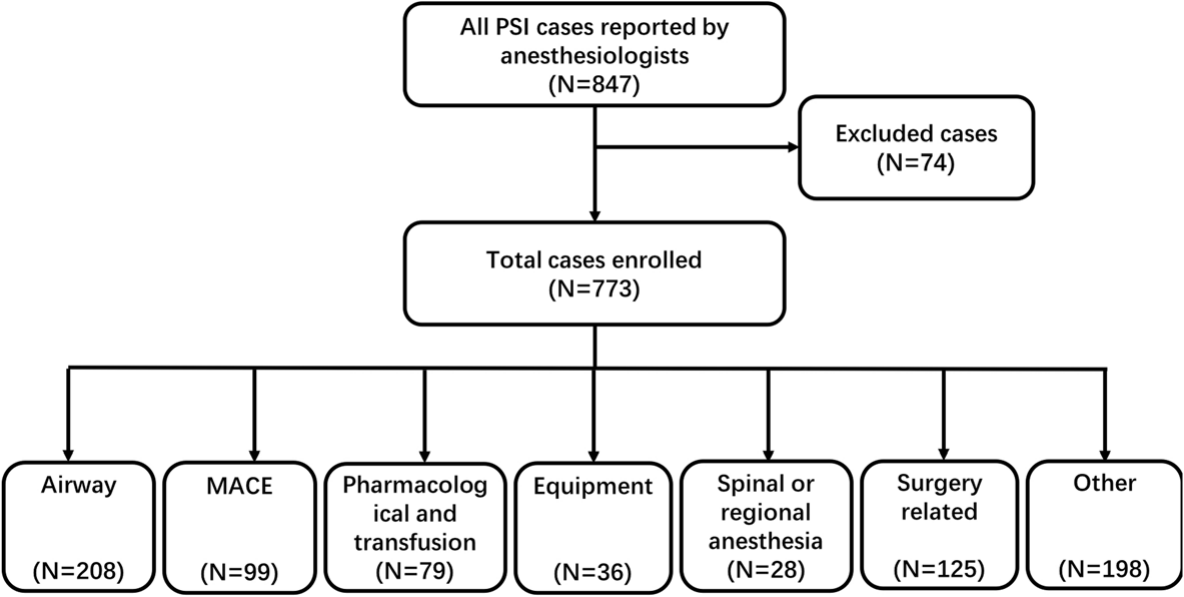
Case enrollment and classification. The figure shows the number of cases reported by anesthesiologists during the 11-year study period and the classification of the final case cohort for analysis. Examples of the different types of incidents are listed below. Airway incidents: intubation failure, bronchospasm or laryngospasm, dental or other oral tissue injury, endotracheal tube dislocation, postintubation hoarseness, aspiration, unplanned secondary intubation, and hypoxemia. MACE: severe hypotension, perioperative acute coronary syndrome, CPR, arrhythmia, cerebral infarction, hydrocephalus, and delirium. Only incidents of heart, brain or vascular origin were classified as this type. Pharmacological and transfusion incidents: drug- or blood product-related events during supply, storage, preparation, and administration; severe adverse drug reactions; anaphylactic reactions; and adverse transfusion events. Equipment incidents: incidents related to monitoring devices, respirators, gas supply systems, anesthesia devices, arterial or venous catheters, or endotracheal tubes. Spinal or regional anesthesia incidents: postdural puncture headache, nerve injury, local anesthetic intoxication, and total spinal anesthesia. Incidents related to surgery: massive bleeding and unplanned secondary operation. Other incidents: problems in multidisciplinary team corporation and communication, anesthesia record problems, and occupational exposure

Data processing included two parts. The first part was incident classification and detail collection. We collected patient sex, age, date, time and place of incident occurrence, type of surgery, and phase of anesthesia when the incident occurred for further group analysis. Incidents were classified into seven types when they were reported: airway incidents, major adverse cardiac and cerebrovascular events (MACE), pharmacological incidents, equipment incidents, spinal or regional anesthesia incidents, incidents related to surgery and other incidents. Examples of the 7 types of incidents are listed below in Fig. 1. For each type of incident, more detailed information was collected.

The second part of the data cleaning was risk assessment. Risk assessment was performed based on the estimated risk of recurrence and estimated consequences for the patient. Then, the incident was automatically classified into four risk categories: extremely high, high, medium and low risk [10]. The reviewers also subjectively classified whether the incidents were preventable, unpreventable or undecided.

### Statistical analysis

Data were stored in a relational structure using Microsoft Office Excel 2016 (Microsoft Corp. 2016, Redmond, Washington, USA). SPSS (IBM SPSS statistics Version 26, Chicago, IL, USA) was used for statistical analysis of the dataset. We described the demographic and basic clinical characteristics of the patients involved in the safety incidents. The “percentage of patients with incidents” for different ASA categories was calculated as the number of patients with reported incidents divided by the total number of patients with the same ASA grade, and the risk ratio with the 95% confidence interval (CI) was estimated using the ASA I as the reference group. A two-sided *P* value less than 0.05 was regarded as statistically significant.

## Results

Of the 847 PSIs voluntarily reported by anesthesiologists from September 2009 to August 2019, 74 cases were excluded because they had already been reported or were not related to anesthesia. In total, 773 cases were enrolled among 452,974 anesthesia care episodes, with an overall PSI reporting incidence of 0.17%. Case numbers of different types of incidents are shown in Fig. 1.

The average age of the 773 patients involved in the incidents was 51.79 (± 31.91), and the median age was 54. Regarding ASA physical status, patients with a worse physical status were more likely to be involved in a PSI (Table 1). Other details related to the patients and the reported incidents are shown in Table 2.

**Table 1:** ASA physical status of patients with reported incidents

ASA	No. of patients with incidents	No. of patients without incidents	% of patients with incidents	Risk ratio (95% CI)
I	78	105,467	0.074	1.00
II	253	118,620	0.213	2.88 (2.24 to 3.72)*
III	89	14,332	0.617	8.40 (6.19 to 11.39)*
IV	37	981	3.635	51.00 (34.31 to 75.80)*
V	5	78	6.024	86.68 (34.16 to 219.91)*

*p < 0.05. CI: confidence interval. ASA: American Society of Anesthesiologists

Since the ASA data of patients without incidents were available only after 2013 due to technological issues (the electronic anesthesia record system in PUMC Hospital was established in 2013, so the ASA status of patients without reported incidents could only be collected after 2013), only data from 2013 to 2019 were used for the analysis related to ASA status

**Table 2:** Demographic variables and other details of the reported incidents

Item (*N*=773)	Detail	Number of incidents (%)
Patient sex	Male	315 (40.8)
	Female	357 (46.2)
	NA	101 (13.1)
Patient age	<18 years old	51 (6.6)
	18 to 65 years old	499 (64.6)
	>65 years old	175 (22.6)
	NA	48 (6.2)
Date of occurrence	Weekday	692 (89.5)
	Weekend	22 (2.8)
	NA	59 (7.6)
Time of occurrence	Working hours (8:00 am-4:00 pm)	445 (57.6)
	Nonworking hours (4:01 pm-7:59 am)	312 (40.3)
	NA	16 (2.1)
Place of occurrence	In the OR (including PACU)	649 (84.0)
	Out of the OR	93 (12.0)
	NA	31 (4.0)
Type of surgery	Elective surgery	620 (80.2)
	Emergency surgery	110 (14.2)
	Labor analgesia	4 (0.5)
	NA	39 (5.0)
Phase of anesthesia when incident occurred	Preinduction	62 (8.0)
	Induction	77 (10.0)
	Maintenance	247 (32.0)
	Emergence	73 (9.4)
	Recovery in PACU	31 (4.0)
	Postoperative period	77 (10.0)
	During spinal or regional anesthesia procedure	45 (5.8)
	NA	161 (20.8)

*OR* operating room, *PACU* postanesthesia care unit

The total number of incidents with available information in each part is not equal to 773 because some of the data were incomplete or missing

### Airway incidents

Twenty-seven percent (208 of 773) of PSIs were airway-related incidents. Sixty-five (31.3%) occurred during intubation, 41 (19.7%) occurred during anesthesia maintenance, 73 (35.1%) occurred during extubation, and 28 (13.5%) incidents were reported after the patient returned to the ward. There was also one patient (0.5%) who had airway obstruction in the OR before anesthesia induction. The most common airway incident categories were bronchospasm or laryngospasm (*N* = 32), postintubation hoarseness (*N* = 28), dental injury (*N* = 20), intubation failure (*N* = 17), intubation delay caused by a difficult airway (*N* = 15), airway obstruction (*N* = 14), and aspiration (*N* = 10). Ninety-six (46.15%) of the patients suffered from airway-related hypoxemia, comprising 34 mild cases (with minimal SpO_2_ ≥ 85% for less than 5 min) and 62 severe cases (with minimal SpO_2_ < 85% or hypoxemia for more than 5 min). Nine patients had bradycardia, and 3 patients had cardiac arrest caused by hypoxemia. We also noticed that 61 patients underwent unplanned secondary intubation for different reasons. Some of those reasons were related to anesthesia procedures, such as airway obstruction or spasm (*N* = 18), hypoxemia after extubation (*N* = 13), residual paralysis of muscle relaxation (*N* = 6), unplanned change of airway maintaining devices (*N* = 3), or endotracheal tube prolapse or dislocation (*N* = 2), while other reasons were anesthesia- (*N* = 3) or patient- or surgery-related reasons (*N* = 16).

### MACE

In total, 72 cardiovascular events and 27 cerebrovascular events were reported as PSIs. However, these events could also be found in other types of incidents. For example, surgical hemorrhage is always accompanied by severe hypotension. As a result, we combined cardiovascular- and cerebrovascular-related incidents in this part of the analysis. The most common types of cardiovascular incidents were hypotension and cardiac arrhythmia, with 205 and 158 incidents each, respectively. Intraoperative blood loss was the most common cause of hypotension (*N* = 112, 54.6%), followed by anaphylactic shock (*N* = 47, 22.9%). Regarding cardiac arrhythmia, sinus tachycardia and bradycardia were most frequently reported, consisting of 49 and 32 incidents, respectively. CPR occurred in 67 patients, with a rate of 1.48 per 10,000 anesthesia episodes. Twenty-four (35.8%) of the CPR cases were cardiogenic, and 43 (64.2%) cases were caused by other reasons, such as surgical hemorrhage and severe hypoxemia due to airway problems.

### Pharmacological and transfusion incidents

Fifty-five pharmacological incidents and 24 transfusion-related incidents were collected from the system. The majority of incidents in the pharmacological category were anaphylactic reactions, among which 20 and 13 were related to antibiotics and blood products, respectively. Other incidents were related to drug supply (*N* = 1), drug storage (*N* = 1), drug preparation (*N* = 4), blood product preparation (*N* = 2), and drug administration (*N* = 4). Six severe adverse drug reactions and 2 cell-saver-related incidents were also reported.

### Other incidents

Incidents occurring more than 5 times among 198 other types of incidents are listed as follows. A total of 143 incidents revealed problems in multidisciplinary team corporation and communication; 12 incidents were related to anesthesia records; and 6 incidents were related to occupational exposure.

### Risk Assessment

Risk assessment data are shown in Table 3. We paid special attention to preventable incidents with extreme or high risk. For airway incidents, 7 were accompanied by secondary intubation, 5 were related to aspiration, and 4 incidents occurred due to unexpected difficult airway intubation failure. For MACE, 10 and 9 were accompanied by hypotension and arrhythmia, respectively. Five patients received CPR. Three patients suffered from perioperative cerebral infarction, and 2 patients were diagnosed with myocardial infarction. Regarding pharmacological incidents, 4 of those incidents were related to the blood distribution procedure. Two incidents occurred during drug preparation and caused incorrect drug administration, resulting in patient harm.

**Table 3:** Risk and preventability assessment for patient safety incidents

Risk/preventability	Preventable (N)	Unpreventable (N)	Undecided (N)
Airway incidents			
Extremely high	2	0	0
High	23	55	6
Medium	47	68	6
Low	1	0	0
MACE			
Extremely high	5	4	4
High	8	19	15
Medium	14	26	0
Low	2	2	0
Pharmacological incidents			
Extremely high	1	0	0
High	8	51	3
Medium	7	2	3
Low	2	1	1

## Discussion

To err is human, and error is unavoidable. PSI reporting can help physicians learn from errors and improve patient safety. The Anesthesia Patient Safety Foundation (APSF) has stated that human errors are one of the most common causes of PSIs. In this article, we did not consider human error as a single incident type for two reasons. First, human error should be considered in every PSI, but the subjectivity of human error identification would influence the reliability of the result. Second, we aimed to identify the systemic factors that could be targeted with an intervention from the department perspective to improve patient safety. With the implementations of improvement measures, human errors would also be avoided to a large extent at the systemic level.

PUMC Hospital is one of the first hospitals to establish a patient incident reporting system in China, and its Department of Anesthesiology has been among the top three departments according to the number of incidents reported for many years. However, the incident reporting of our department during the last 11 years was only 0.17%, which is much less than those reported from developed countries [11–13]. In developing countries such as China, there are numerous reasons contributing to underreporting, including inconvenient reporting systems, inconstant reporting standards, poor safety culture among institutions, fear of punishing action, and inadequate systematic analysis of the reports and feedback [9, 14, 15]. Only a few people work on quality control and patient safety improvement in China, especially in underdeveloped regions. Most physicians have little knowledge of how the reported incidents will be analyzed and how the results will facilitate changes to eventually improve patient safety. Consequently, the phenomenon of underreporting is common in China. Implementation of a better and more convenient PSI reporting system, unification of reporting standards, encouragement of blame-free reporting, periodic summarizing and timely feedback of PSI data to the public may help increase the PSI reporting rate.

Airway incidents were the most common type of incidents reported and were the top concern of anesthesiologists. This is in accordance with other anesthesia-related incident research [11] but different from the incident composition reported by other departments, such as intensive care units (ICUs) [16]. ICU incident analysis has revealed that airway incidents cause more harm for patients than other types of incidents [17], so anesthesiologists should pay more attention to airway incidents.

Pharmacological incidents are always associated with harm for patients [18]. Runciman and colleagues reported that 36% of anesthesia-related incidents were associated with adverse drug events [19]. Webster and colleague found that one drug administration error was reported for every 133 anesthetics [20]. However, only 79 pharmacological incidents (including transfusion-related incidents) were reported in our PSI system, which is much less than the PSI reporting rate in other studies. We considered that the main reason for this result was that many events were not reported because the doctor did not notice that a PSI had occurred or because there is a misconception that such events do not cause severe patient harm such that reporting is not necessary. Therefore, the reporting incidence was far from satisfactory. Medication error in anesthesia practice is unavoidable and could cause severe harm to patients. Consequently, far-reaching changes are needed on improving safety of drug preparation and administration, and the culture of reporting medication errors on PSI reporting system should be cultivated.

Risk assessment is useful for helping physicians determine the types of incidents that are harmful for patients but preventable, enabling intervention to be performed from the department perspective. Using airway incidents as an example, we found from our analysis that among those preventable incidents with extreme or high risk, 7 were accompanied by secondary intubation. Therefore, we reported all secondary intubation cases during the daily morning shift in detail so every physician could learn from these cases and pay more attention to them in their clinical work. We also noticed that many cases were related to unexpected difficult airway intubation failure. Therefore, we conducted difficult airway management training for physicians to improve their mastery skills.

We also identified some common types of incidents in our analysis that warrant further investigation. For example, 28 postintubation hoarseness incidents were reported, among which 25 were caused by arytenoid dislocation (AD). AD is a rare but severe complication after general anesthesia with endotracheal intubation. This complication frequently appeared in our incident reporting system and had already attracted our attention. We conducted a case-control study and identified that AD was associated with prolonged operative time and that an intubation stylet appeared to protect against AD [21]. Consequently, our department encouraged anesthesiologists to use an intubation stylet, especially for patients who underwent long-term surgery.

Standard operating procedures (SOPs) could be implemented to provide physicians with guidance for addressing clinical situations that may cause PSIs. For instance, most of the pharmacological incidents reported in our study were anaphylaxis reactions, which often have a quick onset and can cause serious threats to life if not treated rapidly and correctly. Therefore, our department has implemented an SOP for intraoperative anaphylaxis, which not only reminds anesthesiologists to pay attention to drug allergy prevention but also guides them in treatment and resuscitation when anaphylaxis occurs. We had already implemented several SOPs (e.g., difficult airway management, bronchospasm and laryngospasm, postintubation hoarseness, anesthesia-related dental injury, aspiration) based on the results of PSI analysis. More SOPs should be introduced in the future, and the effectiveness of SOPs should be further evaluated.

**Fig. 2:**
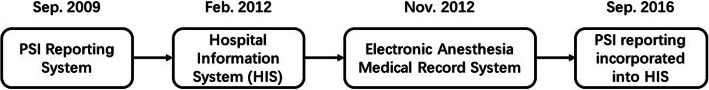
Development of PSI reporting system and electronic medical record system in PUMC HOSPITAL. The figure shows the timetable of the PSI reporting system and electronic medical record system (including the hospital information system and electronic anesthesia medical record system) in PUMC Hospital

The incorporation of electronic medical records has highly impacted PSI reporting, especially influencing the quality of further data collection. As an example, our hospital (Fig. 2) established a PSI reporting system in September 2009. PSIs were inefficiently collected artificially by the department of medical affairs. With the implementation of the Hospital Information System (HIS) and the Electronic Anesthesia Medical Record System in 2012, the quality of data collection greatly improved. The PSI reporting system was incorporated into the HIS in September 2016, which further improved the integrity of the database. We believe that PSI reporting will be more convenient and reliable with the development and improvement of electronic medical records in the future.

This study also has some limitations. First, the freeform text incident data were subjective and incomplete. This was a common problem with all PSIs, so misunderstanding and imperfection were unavoidable to a large extent. Both the lack of an electronic medical record system in the first three years and lack of an electronic reporting system between 2009 and 2016 caused difficulty in data collection. To compensate for the deficiency as much as possible, we supplemented incident information by reading patients’ records and tried our best to ensure the validity and reliability during data cleaning as described above. Second, our study was a single-center study, and the phenomenon of underreporting was common. Furthermore, nonroutine events without patient injury or even with mild patient physiological disturbances may not be reported, although these events were also important for guiding organizational patient safety improvement interventions [22]. Consequently, the result may not reflect the complete situation. Underreporting is unavoidable, but our hospital has taken many measures to increase the reporting rate. For example, our hospital has specifically assigned administration staff to manage those reported incidents and provide feedback to the related department and individual. The hospital also provides financial incentives to encourage incident reporting. Our department has a quality control group and periodically analyses PSI information and shares summary reports with the entire department to provide feedback. Therefore, our reporting rate is relatively high in China. More complete incident reporting systems should be established, and better incident reporting cultures should be cultivated in developing countries. Large, multicenter trials may be needed, and more attention should be paid to better summarizing incidents and making the data more valuable in the future.

## Conclusions

We analyzed 847 PSIs voluntarily reported by anesthesiologists within eleven years in a Chinese teaching hospital. The reporting rate was only 0.17%, reflecting that underreporting is still common in China. Airway incidents constitute the majority of incidents, which is in accordance with other developed countries. The importance of summarizing and utilizing anesthesia incidence data should be scrutinized. Measures and SOPs should be implemented from the department or a higher organizational perspective based on the PSI analysis results, such as the PSI events summary and feedback.

## Data Availability

The datasets generated and analyzed during the current study are available from the corresponding author on reasonable request.

## Abbreviations

WHO: World Health Organization
PSI: patient safety incident
NRLS: National Reporting and Learning Service
PUMC: Peking Union Medical College
MACE: major adverse cardiac and cerebrovascular events
APSF: Anesthesia Patient Safety Foundation
ICU: intensive care unit
AD: arytenoid dislocation
SOP: standard operating procedures
HIS: Hospital Information System
